# Total Hemoglobin Trajectories from Pregnancy to Postpartum in Rural Northeast Brazil: Differences between Adolescent and Adult Women

**DOI:** 10.3390/ijerph19073897

**Published:** 2022-03-25

**Authors:** Raí Nabichedí da Silva, Catherine M. Pirkle, Tetine Sentell, Nicole Kahielani Peltzer, Yan Yan Wu, Marlos R. Domingues, Saionara M. A. Câmara

**Affiliations:** 1 Postgraduate Program in Public Health, Faculty of Health Sciences of Trairi, Federal University of Rio Grande do Norte, Santa Cruz 59300-000, Brazil; rai.nabichedi@gmail.com; 2 Office of Public Health Studies, Thompson School of Social Work & Public Health, University of Hawaii at Mānoa, Honolulu, HI 96822, USA; cmpirkle@hawaii.edu (C.M.P.); tsentell@hawaii.edu (T.S.); npeltzer@hawaii.edu (N.K.P.); yywu@hawaii.edu (Y.Y.W.); 3 Postgraduate Programme in Physical Education, Federal University of Pelotas, Pelotas 96055-630, Brazil; marlosufpel@gmail.com

**Keywords:** hemoglobin, pregnancy, postpartum, longitudinal studies, mother child health, anemia, prenatal care

## Abstract

This study examines total hemoglobin (THB) trajectories during pregnancy and postpartum and associated factors among adolescents and adults from a low-income community. This is an observational, longitudinal study, part of the Adolescence and Motherhood Research (AMOR) project, performed between 2017 and 2019 in the Trairi region of Rio Grande do Norte state, Brazil. The THB levels of 100 primigravida adolescents and adults were monitored up to 16 weeks of gestation, in the third trimester, and 4–6 weeks postpartum, along with socioeconomic characteristics, anthropometrics, and health-related variables. Mixed-effect linear models evaluated the trajectories of THB and the associated factors. THB levels decreased between first and second assessments and increased between the second and postpartum assessments. For the adolescent cohort, the rebound in THB concentration between the third trimester and postpartum was not enough to make up for the initial losses, as occurred in the adult cohort. For the adult group, higher THB levels were associated with pregnancy planning and good self-rated health. Race was marginally associated to THB levels, with black/brown women presenting higher concentrations in the adolescent and lower concentration in the adult group. Special attention to prenatal care among pregnant adolescents should consider their higher risk of anemia and its negative effects.

## 1. Introduction

During pregnancy, physiologic iron demands increase to support blood volume expansion and total red blood cell mass, allowing fetal hematopoiesis and adequate blood supply to the fetus [1]. These adjustments occur during the first half of pregnancy and contribute to reduced iron stores, lower hemogloblin levels, and anemia at the end of pregnancy [2]. Generally, hemoglobin levels drop from the first to third trimester of pregnancy [3] and return to pre-pregnancy levels postpartum [4]. However, when iron stores drop drastically during pregnancy, complications like gestational anemia [5], insufficient fetal weight gain [6], and maternal and fetal mortality [7] can arise.

Hemoglobin levels variate during pregnancy across socioeconomic conditions, lifestyles, and health behaviors [8,9]. In Brazil, a study analyzing medical records of low-risk pregnant women found that hemoglobin levels were lower in poorer northern regions of the country compared to wealthier southern regions [9]. Childbearing age also affects hemoglobin levels during pregnancy [10]. During adolescence, there is a greater physiological need for hemoglobin due to increased erythrocytic mass for rapid somatic growth [11] and menstruation onset [2]. Pregnancy imposes additional hemoglobin requirements [2,6], rendering pregnant adolescents vulnerable to anemia and adverse maternal and child health consequences, like intrauterine growth retardation [11] and low birth weight [6].

In Brazil, approximately 419,252 adolescents give birth per year [12]. Although progress has been made in recent decades to reduce adolescent fertility, Brazil still has higher rates of adolescent pregnancy compared to other Latin American and Caribbean countries [13]. Despite risks to mother and child, there are few studies about hemoglobin trajectories of pregnant adolescents, particularly from low and middle-income settings like Brazil.

Hemoglobin levels are regularly measured in clinical practice [14]. Tracking variations in these levels can explain clinically significant changes in the health status of pregnant women, especially if hemoglobin levels drop critically [15]. Understanding how hemoglobin levels vary during pregnancy and postpartum will help direct public health policies to reduce adverse effects of hemoglobin drops on pregnant adolescents. The aim of this study is to examine the variation in hemoglobin trajectories during pregnancy and postpartum and associated factors among adolescents and adults from a rural low-income area of Northeast Brazil.

## 2. Materials and Methods

### 2.1. Design and Study Location

This is an observational, longitudinal study that is part of the pilot project Adolescence and Motherhood Research (AMOR) [16]. The AMOR project was designed to test the feasibility of a research project that could test the hypothesis that adolescent pregnancy increases the risk of chronic conditions and mobility loss over time. This paper analyzes secondary data from this cohort study. Data were collected between July 2017 and January 2019 in five municipalities of the Trairi region, a rural region of the Rio Grande do Norte State, Brazil: Santa Cruz, Lajes Pintadas, Tangará, Campo Redondo and São Bento do Trairi. In AMOR, pregnant adolescents and adults were evaluated at three time periods: once during the first 16 weeks of gestation, once between 27 and 40 weeks, and once more between 4 and 6 weeks postpartum.

### 2.2. Population and Sample

The research participants were pregnant adolescents (13 to 18 years) and adults (23 to 28 years) in their first pregnancy. Initially, the sample consisted of 50 pregnant women in each age group, totaling 100 participants at baseline. The baseline sample size for AMOR project was established at 100 participants which provided sufficient statistical power to assess the validity of epidemiological instruments [17], one of the main objectives of the AMOR project. Participants were included if they were in the selected age range, in their first pregnancy, and residing in one of the five municipalities where this study was conducted. At baseline, women were excluded if they had: chronic conditions (hypertension, diabetes, HIV infection) diagnosed before pregnancy or during their first prenatal visit, as well as those who used medications that could interfere with the blood parameters, such as antidepressants and anxiolytics. One participant was excluded for using antidepressant medication.

### 2.3. Instruments

The AMOR study questionnaire was prepared by the project researchers. The baseline questionnaire addressed aspects related to socioeconomic conditions, social networks, health status, quality of life, food security, lifestyle, health behaviors, and reproductive history. Follow-up assessments collected information about the third trimester of pregnancy and the postpartum period, including prenatal care and health literacy in the third trimester assessment and information about delivery and postnatal care in the postpartum assessment. The follow-up questionnaires only included questions about aspects that may have changed from the baseline questionnaire. Baseline and follow-up assessments took 1.5 h and 1 h respectively. The details about how and when these study variables were collected are presented below.

### 2.4. Measurements

During the three assessments, we performed physical assessments and collected blood samples. The description of how the measures included in this study were taken are described below.

#### 2.4.1. Total Hemoglobin Concentration (THB)

THB concentrations were determined from dried blood spots (DBS). Five drops of capillary blood were collected from each participant on a filter paper card (Whatman^®^ 903) which remained at room temperature for 4 h to dry and then stored at −80 °C. Subsequently, the samples were sent on dry ice to the University of Washington Department of Laboratory Medicine and Pathology Biomarker Laboratory for THB analysis (Seattle, WA, USA).

The THB concentrations of each participant, as measured from the DBS, were determined by a modified version of the alkaline haematin D-575 method [18]. In brief, 25 µL of eluent from a 3.2 mm diameter DBS punch in 200 µL dd/diH2O was mixed with 100 µL of THB Reagent (2.5% Triton X-100, 97.5% 0.1 M NaOH; Sigma-Aldrich, St. Louis, MO, USA), incubated at room temperature for 15 min in the dark, and the absorbance of each well was then measured at 405 nm (Synergy HT, BioTek, Winooski, VT, USA). THB of quality control DBS-matched gold-standard venous blood samples were measured on a Sysmex America XE-5000 Automated Hematology Analyzer (Mundelein, IL, USA). The THB was converted in blood-equivalent THB using a simple linear regression considering the THB of 48 quality control blood samples as gold-standard [19]. The reportable range of DBS blood-equivalent THB was 5.0–18.4 g/dL.

#### 2.4.2. Weight and Height

Weight and height were collected in all three waves of data collection using a digital scale (Omron^®^, model HN289) and stadiometer (Seca^®^, model 213), respectively.

### 2.5. Procedures

Participants were recruited from the primary health-care units of participating cities. Health professionals from all units, mostly nurses, were contacted weekly by the project recruiters and informed about potential participants. Participants were later contacted by the researchers and invited to participate. Before initiating data collection, the participants received detailed explanations about the study procedures, as well as the legal guardians of those under 18 years old. Consent forms were given after detailed explanations about study procedures. Data were collected by trained interviewers through an electronic questionnaire using the Qualtrics software [20] and a tablet device (Samsung, Galaxy model A 10.1). Assessments were conducted in the primary health units and in the Faculty of Health Sciences of Trairi, campus of Federal University of Rio Grande do Norte located in the city of Santa Cruz, after prior scheduling with the participants and following a standardized protocol [16]. Participants unable to reach one of these locations, due to transportation or health challenges, were interviewed in their homes.

The sociodemographic variables were collected in the baseline questionnaire. Age in years was collected using national identity cards. Race was self-reported and the participants were classified into white and black/mixed-race per standard Brazilian classifications [21]. Marital status was self-reported and classified into married/common-law or single/separated/divorced.

We evaluated socioeconomic status through variables related to education, income, and childhood conditions. Education was collected by asking participants how many full years of schooling they had completed. Income sufficiency was evaluated by asking women to classify the extent to which her financial situation met their needs, with the response options: very sufficient, suitable, or not sufficient/not at all. *Bolsa Família* is a Brazilian direct cash transfer program to low-income families, which contributes to decrease poverty and inequality [22]. Participants reported if someone from their family received *Bolsa Família*. Socioeconomic status during childhood was collected by asking the participant about her economic conditions during her first 10 years of life: good, moderate, or poor. Participants with lower years of schooling, reporting that their financial situation is not sufficient to meet their needs, having a family member receiving *Bolsa Família*, and referring poor socioeconomic conditions during childhood can be considered as having lower socioeconomic status than the others.

We evaluated self-rated health during all three assessments. Participants classified their current health status according to a scale ranging from 0 (bad health) to 100 (good health). Those who perceived their health in the lowest 20th percentile of the sample (score < 70) were classified with moderate/poor health. All others were classified with good self-perceived health.

The number of prenatal appointments was self-reported in the postpartum assessment. This variable was introduced as an indication of adequacy of prenatal care [23]. Of note, participants were also asked if they received or bought iron pills during pregnancy. Since only one participant answered no, iron supplementation was not considered in the analyses.

The participants were asked about whether the pregnancy was planned in the baseline assessment, and the answers were categorized as yes or somewhat/not at all.

Food security was assessed at the baseline through a questionnaire based on the validated Latin American and Caribbean Food Security Scale [24]. Nine questions asked about the participant’s food security experience in the past three months, for example, whether or not she worried about running out of food or skipped a meal because of a lack of food. Each question had as response options: “almost every day”; “a few days, but not all”; “rarely, maybe twice”; and “almost never or never”. Food insecurity was defined if an affirmative answer was given to any of the 9 questions [24].

The age of menarche was also collected at the baseline through the question “At what age did you have your first menstrual period?”.

Type of delivery (vaginal or cesarian section) and breastfeeding (y/n) were collected through self-report at the postpartum assessment.

### 2.6. Ethics

The project was approved by the Research Ethics Committee of the Faculty of Health Sciences of Trairi and the National Commission for Research Ethics (approval number 1.902.815) and by the Institutional Review Board of the University of Hawai’i at Mānoa. All adult participants signed the consent form and the adolescents signed the assent form, following the signed consent of a legal representative, according to Brazilian ethical regulations.

### 2.7. Data Analysis

Data were analyzed with the software SPSS^®^ version 20.0 and the software R 3.5.1. Descriptive statistics were performed to characterize the sample according to age group. For the analysis of quantitative variables, the Mann–Whitney test was used in view of the non-normal distribution of the data, identified by the Shapiro–Wilk test and the Chi-square test was used for categorical variables. The participants’ characteristics at baseline were compared with those retained until the third evaluation. In all stages, 95% confidence intervals (CI) and 5% level of significance were considered.

We also compared the means of THB for categorical variables using Student’s *t*-test or ANOVA test with Tukey’s post hoc (hemoglobin concentrations in the sample followed normal distribution curves). The correlations between the quantitative variables (age, years of schooling, number of prenatal appointments, age at menarche, weight, and height) and hemoglobin in the three evaluations were assessed using Spearman’s and Pearson’s tests.

To examine if the changes in THB at follow-up visits differed by age group, mixed-effects models were performed with age-by-time interaction where time was treated as categorical variable. Next, a stepwise regression strategy was applied to identify variables that were associated with THB (with age-by-time interaction retained in the model). Although not statistically significant, due to descriptive findings and the strength of the coefficients, age-by-time interaction term was maintained. An interaction term between race and age group was also added to the models to verify if the mean THB for age group varied by race. Finally, to identify variables associated to THB variation during pregnancy and postpartum in each age group, we performed stratified analysis by age following the same procedures.

## 3. Results

**Figure 1:**
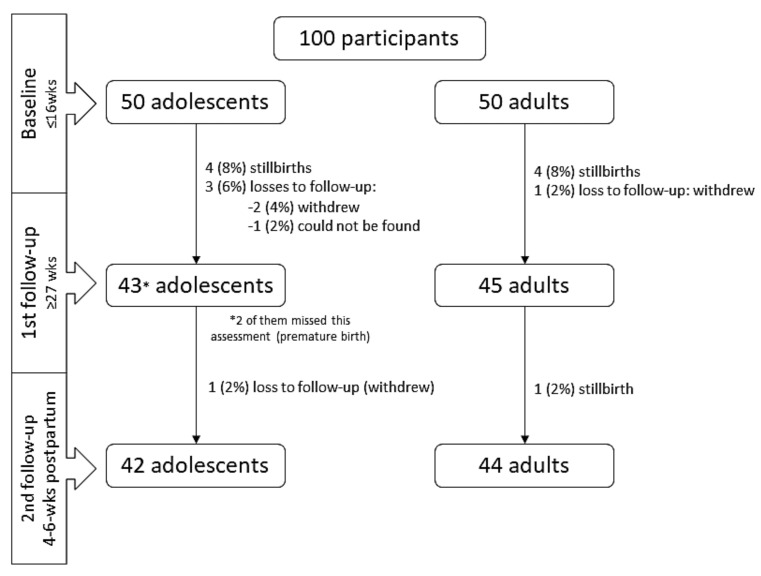
Flowchart of the sample by age group of AMOR project in the Trairi region, Rio Grande do Norte, Brazil, 2017–2019. * Two participants were not assessed in the 1st follow-up because of premature birth but they returned for the 2nd follow-up.

Figure 1 depicts the number of participants from baseline to postpartum. Eight participants were lost to follow-up in the cohort of adolescents and six from the adult cohort (Figure 1). There were no statistically significant differences in participant characteristics at baseline and those followed to six-weeks postpartum (data not shown).

Table 1 presents the characteristics of the sample. There were statistically significant differences between the adolescents and adults in the years of schooling and bodyweight, with the adult cohort presenting higher values, as expected. A marginally higher proportion of adolescents reported receiving Bolsa Família and a marginally higher proportion of adults were married and food secure.

Table 2 presents the THB means according to the independent variables at the three assessments. At baseline, significantly lower values of THB were observed among those who reported very sufficient incomes when compared to the others, as well as among those who reported poor health status. The mean THB was also statistically lower among those who reported being single, separated or divorced in the second evaluation and was statistically higher among those who reported not receiving Bolsa Família during the postpartum evaluation. There were marginal associations for those who planned their pregnancies at baseline and during the third evaluation. Those that reported having planned their pregnancies had higher THB at both assessments.

Table 3 shows the correlation between THB and the quantitative independent variables. There was no statistically significant difference between THB and the variables years of schooling or prenatal appointments. Those with higher weights and heights had higher THB concentrations in the third trimester evaluation.

**Figure 2:**
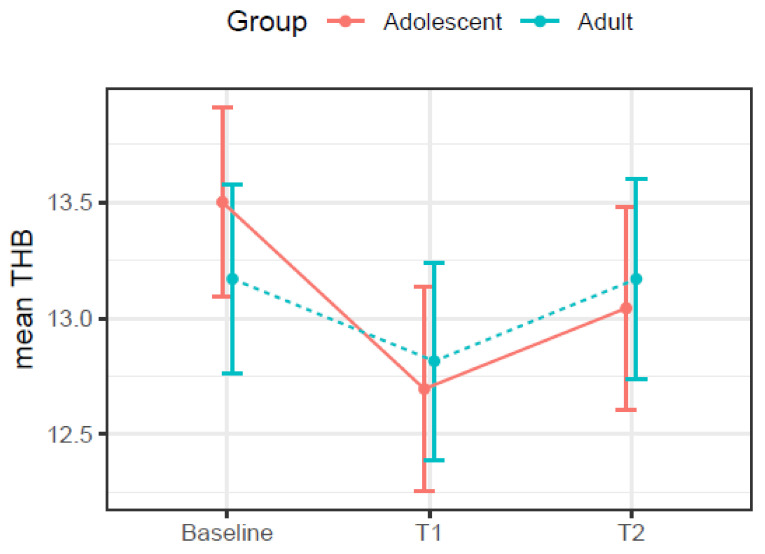
THB variation from the first 16 weeks of pregnancy (baseline) to the third trimester (T1) and the 4 to 6 weeks postpartum (T2) for adolescents and adults. Trairi Region, Rio Grande do Norte, Brazil, 2017–2019.

Table 4 shows the results of the multivariate mixed-effects regression for independent variables on the THB trajectory. At baseline, adolescent participants started with a statistically significant higher mean THB when compared to the adult group. Between baseline and the second evaluation, the adolescent group experienced a significant drop in THB (−0.86 g/L), which rebounded 0.46 g/L between the 2nd and 3rd evaluation but did not return to the mean baseline level. This contrasted with the adults (Figure 2), who, similar to the adolescents, experienced a drop in mean THB between baseline and 2nd evaluation, but mean THB returned to baseline levels between the 2nd and 3rd evaluation.

Women who reported planning their pregnancies had significantly higher THB levels across all assessments. Those who reported poor self-perceived health status had marginally lower THB levels. Age-by-race interaction was also statistically significant. The mean THB among white adolescents was 0.55 g/L lower than non-white adolescents, while the mean THB among white adults was 0.56 g/L higher than non-white adults.

**Figure 3:**
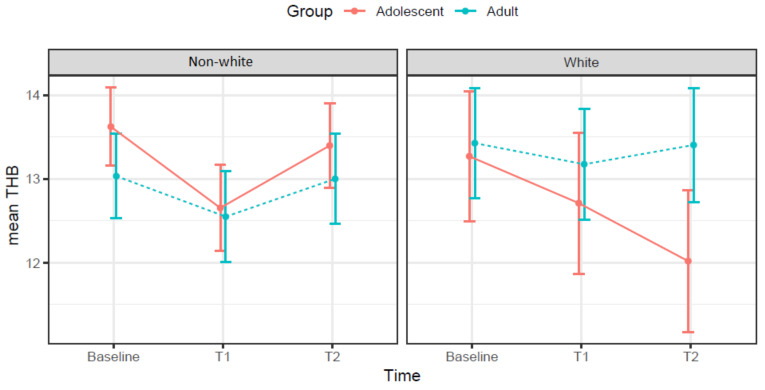
THB variation from the first 16 weeks of pregnancy (baseline) to the third trimester (T1) and the 4 to 6 weeks postpartum (T2) for white and non-white adolescents and adults. Trairi Region, Rio Grande do Norte, Brazil, 2017–2019.

Figure 3 presents the results for the interaction between age and race for the THB level in each wave of data collection. While the THB trajectories for non-white adolescents are similar to adults from both races, i.e., reduction in the third trimester and rebound in the postpartum, the results for white adolescents show that their THB levels continued declining during the postpartum assessment.

Table 5 presents the mixed-effects model for independent variables in relation to THB trajectory stratified by age group. The results indicate that, for adolescents, none of the independent variables presented significant associations with THB levels. For adults, there was a statistically significant association between pregnancy planning and THB. Women who reported planning their pregnancies had higher THB concentration, which were, on average, 0.57 g/L higher than women who did not fully plan their pregnancies. Moreover, there was a marginally significant association between self-rated health with THB for the adult group. Those classified with poor self-perceived health status had lower THB (−0.58 g/L, *p* = 0.075) compared to those classified with good health status.

There are important THB variation differences between both age groups during pregnancy and postpartum. The adults experienced a milder drop in THB between the first and third trimesters (−0.40 g/L), returning to approximately the same initial THB concentration (mean difference between T2 and baseline of −0.03). In addition, the intercept for adults was 0.79 g/L lower than for adolescents. For the adolescents, the THB dropped significantly between the first and the second assessments (−0.86 g/L) and had a slight recovery between the second and third (0.38 g/L). Thus, for the adolescent cohort, the rebound in hemoglobin concentration between the third trimester and postpartum was not enough to make up for the initial losses and, by six weeks postpartum, they had, on average, a hemoglobin concentration 0.48 g/L lower than when they were in the first weeks of pregnancy. Figure 2 shows a visual representation of the THB variation in each group.

## 4. Discussion

This study evaluated THB trajectories during pregnancy and postpartum along with associated factors in adolescents and young adults. We observed decreased THB levels from the first 16-weeks of pregnancy to the third trimester and increased from the third trimester to the 4–6 weeks postpartum for both groups. This is consistent with the literature [4,25]. However, the groups differed in relation to the THB trajectories. For the adolescent cohort, the rebound in THB concentration between the third trimester and postpartum did not make up for initial losses. At 6-weeks postpartum, adolescents had lower THB concentrations compared to early pregnancy levels. For the adult group, the initial THB drop was similar to the rebound observed between the third trimester and postpartum. Higher THB levels were marginally associated with pregnancy planning and self-rated health among the adults. Race was marginally associated to THB levels for both groups but with different directions of association.

According to our results, adolescents started pregnancy with higher mean THB than adults. Since adolescence is a phase of rapid somatic growth [10], there is possibly a greater physiological demand for oxygenation of muscles and tissues [2] that leads to an increase in THB levels among adolescents. Moreover, it is possible that fewer adolescent periods lead to less lifetime hemoglobin losses.

During pregnancy, the fetus competes with the mother for sustenance [26]. Competing demands from pregnancy and adolescent body development may make teenagers more vulnerable to iron deficiency and anemia [10]. Thus, the higher demand for nutrients among adolescents, compared to adults, may explain the greater reduction in hemoglobin levels among adolescents during pregnancy, as we observed.

After pregnancy, plasma volume decreases which raises hemoglobin levels [25] and hemoglobin concentrations postpartum return to similar levels as early pregnancy. However, based on our results, this return was more compensatory for adults than adolescents. The greater physiological demand required by adolescence suggests that although hemoglobin levels return, it is not proportional to the drop observed during pregnancy.

We also found that self-reported health was marginally associated with the trajectories of THB, with higher levels among those with good self-reported health. Self-reported health is strongly related to morbidity, mortality, and longevity in different population subgroups [27]. In pregnant women, poor self-reported health is associated with infection, anemia, hypertension, and severe bleeding during pregnancy and postpartum hemorrhage [28]. Our study findings support that self-reported good health status is associated with better health. Pregnant women reporting poorer health status should be screened further for total hemoglobin concentrations and other physiological dysregulations.

Pregnancy planning was also associated with THB trajectories. A previous study from Finland reported better biomarker results among women who planned their pregnancies compared to those with unplanned pregnancies [29]. The authors found that glycated hemoglobin levels, used to identify diabetes, were significantly lower before and throughout pregnancy among women with planned pregnancies. In addition, planned pregnancies are associated with significantly less pregnancy and childbirth related risks [29]. This evidence suggests that greater attention to one’s health before pregnancy may be associated with better health outcomes overall [30]. Thus, it is possible that women who planned their pregnancies may have healthier life habits, like taking supplemental iron, translating into higher THB levels.

Regarding race, in the present study, the results for both age groups are contradictory. While being non-white was marginally associated with higher THB levels for adolescents, non-white participants presented lower THB levels among the adults. The results for the adult group are similar to those from other previous studies, which have also observed that non-white pregnant women have lower hemoglobin and hematocrit levels when compared to white peers [31,32]. Furthermore, the THB trajectory during pregnancy and postpartum in both race groups among adolescents and adults has different directions. Different from the other groups, white adolescents continued to have a decrease in THB concentration after childbirth. These results warrant further research.

### Strengths and Limitations

The present study has limitations. Although the small sample is sufficient for longitudinal analyses of biomarker concentrations, it has limited power in interaction analyses, especially after stratification by age. In addition, the small sample size prevented comparing the proportions of anemia between both age groups. The study took place in a specific area in northeastern Brazil and the increased fertility rates among very young adolescents and the severe lack of studies explaining these trends in this context justified the choice of this area for the AMOR project [16]. However, these results can only be generalized to other rural, low-income regions of Brazil. In addition, although the data collected indicate that almost all participants were taking prenatal iron pills, we could not measure adherence to the supplementation during pregnancy. It is well established that iron supplementation is effective in increasing iron reserves and hemoglobin synthesis to prevent anemia during pregnancy [9]. Finally, we only measured total hemoglobin values and did not collect data on serum ferratin, which directly measures iron deficiency.

## 5. Conclusions

Pregnancy during adolescence is a public health concern and this study presents novel results showing how hemoglobin trajectories during pregnancy and childbirth differ among adolescents and adults. The results observed in the limited return of hemoglobin levels for adolescents highlight the importance of implementation of public health policies for this population. It is necessary to strengthen local strategies to guide health professionals on the importance of increasing attention to prenatal recommendations, especially for adolescents and those who did not plan their pregnancies. Recommending a balanced diet rich in iron for all women, even those who are not intending to become pregnant, is also important. Finally, it is necessary to emphasize that further research is needed to evaluate whether the results found here are similar in other regions of Brazil.

## Figures and Tables

**Table 1:** Baseline sample characteristics (N = 100). Trairi region, Rio Grande do Norte, Brazil, 2017–2019.

	Adolescent (*n* = 50)	Adults (*n* = 50)	*p* Value
	Median (IQR) or *n* (%)		
Years of schooling	9.00 (3)	12.00 (4)	<0.001
Race			0.198
White	13 (26.0%)	19 (38.0%)	
Mixed race/black	37 (74.0%)	31 (62.0%)	
Income sufficiency ^a^			0.715
Very sufficient	9 (18.0%)	12 (24.5%)	
Suitable	29 (58.0%)	27 (55.1%)	
Not at all	12 (24.0%)	10 (20.4%)	
*Bolsa família* ^a^			0.052
Yes	39 (78.0%)	28 (56.0%)	
No	11 (22.0%)	21 (42.0%)	
Marital status			0.096
Married/Common law	35 (70.0%)	42 (42.0%)	
Single/Separated/Divorced	15 (30.0%)	8 (16.0%)	
Childhood SES ^a^			0.363
Good	23 (47.9%)	17 (34.0%)	
Moderate	19 (39.6%)	26 (52.0%)	
Poor	6 (12.5%)	7 (14.0%)	
Weight	54.99 (10.32)	61.49 (10.37)	0.002
Height	156.91 (5.29)	159.17 (6.20)	0.052
Self-perceived health			0.790
Good	42 (84.0%)	41 (82.0%)	
Poor	8 (16.0%)	9 (18.0%)	
Number of prenatal appointments ^b^	9.00 (3)	9.00 (2)	0.602
Planned pregnancy			0.420
Yes, entirely	20 (40.0%)	24 (48.0%)	
Somewhat/Not at all	30 (60.0%)	26 (52.0%)	
Food security			0.068
Yes	9 (18.0%)	17 (34.0%)	
No	41 (82.0%)	33 (66.0%)	
Age at menarche ^c^	12.00 (1)	12.00 (3)	0.871

^a^: 01 missing value. ^b^: 19 missing values. ^c^: 02 missing values. SES: Socioeconomic Status. IQR: interquartile range.

**Table 2:** Means (SD) of THB according to the independent variables.

	Baseline (*n* = 100)	T1 (*n* = 88)	T2 (*n* = 86)
Age group	**Mean (SD)**		
Adolescents	13.50 (1.32)	12.70 (1.65)	13.04 (1.51)
Adults	13.17 (1.25)	12.82 (1.27)	13.17 (1.69)
*p* value ^a^	0.209	0.720	0.723
Race			
White	13.35 (1.44)	12.96 (1.19)	12.86 (1.21)
Mixed race/black	13.33 (1.23)	12.66 (1.57)	13.23 (1.75)
*p* value ^a^	0.934	0.327	0.317
Income sufficiency ^b^			
Very sufficient	12.34 (1.01)	12.75 (1.14)	13.11 (1.39)
Suitable	13.61 (1.28)	12.90 (1.68)	13.23 (1.78)
Not at all	13.59 (1.13)	12.45 (1.10)	12.81 (1.35)
*p* value ^c^	<0.001 **	0.502	0.616
*Bolsa família* ^b^			
Yes	13.38 (1.35)	12.84 (1.50)	12.85 (1.49)
No	13.23 (1.18)	12.62 (1.40)	13.65 (1.72)
*p* value ^a^	0.591	0.517	0.031
Marital status			
Married/Common law	13.38 (1.33)	12.91 (1.36)	13.06 (1.60)
Single/Separated/Divorced	13.16 (1.16)	12.03 (1.75)	13.38 (1.57)
*p* value ^c^	0.489	0.033	0.531
Childhood SES ^b^			
Good	13.01 (1.14)	12.75 (1.40)	12.75 (1.66)
Moderate	13.49 (1.28)	12.68 (1.54)	13.52 (1.68)
Poor	13.62 (1.54)	13.05 (1.45)	12.75 (0.92)
*p* value ^c^	0.152	0.738	0.090
Self-perceived health			
Good	13.48 (1.28)	12.74 (1.51)	13.11 (1.64)
Poor	12.64 (1.14)	12.83 (1.07)	13.11 (1.18)
*p* value	0.014 ^a^	0.864	0.992
Planned pregnancy			
Yes, entirely	13.61 (1.45)	12.80 (1.40)	13.48 (1.64)
Somewhat/Not at all	13.11 (1.11)	12.74 (1.51)	12.83 (1.51)
*p* value ^a^	0.058	0.845	0.061
Food security			
Yes	12.96 (1.23)	13.21 (1.37)	12.79 (1.78)
No	13.47 (1.29)	12.62 (1.46)	13.20 (1.53)
*p* value ^a^	0.085	0.107	0.314
Type of delivery ^d^			
Vaginal	13.34 (1.32)	12.91 (1.49)	12.87 (1.41)
Cesarean	13.32 (1.37)	12.58 (1.43)	13.33 (1.74)
*p* value ^a^	0.951	0.301	0.184
Breastfeeding ^e^			
Yes	13.40 (1.35)	12.76 (1.44)	13.05 (1.55)
No	12.65 (0.77)	13.03 (0.96)	13.68 (1.87)
*p* value ^a^	0.277	0.301	0.434

^a^: *p* value for Student’s *t* test. ^b^: 01 missing value. ^c^: *p* value for ANOVA test. ^d^: 16 missing values in the baseline, 15 missing values in the T1 and 14 missing values in the T2. ^e^: 18 missing values in the baseline, 17 missing values in the T1 and 16 missing values in the T2. ** Very sufficient< Suitable; Very sufficient< not at all. THB: Total hemoglobin. SES: Socioeconomic Status.

**Table 3:** Correlation between THB and independent variables.

	Baseline (*n* = 100)		T1 (*n* = 88)		T2 (*n* = 86)	
	Coefficient	*p* ^a^	Coefficient	*p* ^a^	Coefficient	*p* ^a^
Years of schooling	−0.087	0.395	−0.035	0.748	−0.046	0.673
Weight	0.094	0.357 ^b^	0.218	0.043 ^b^	−0.860	0.429 ^b^
Height	0.093	0.363 ^b^	0.226	0.036 ^b^	0.049	0.651
Number of prenatal appointments	−0.118	0.301	−0.084	0.460	−0.006	0.960
Age at menarche	0.044	0.670	0.087	0.423	0.015	0.892

^a^: *p* value for Spearman’s test. ^b^: *p* value for Pearson’s test. THB: Total hemoglobin.

**Table 4:** Multivariate mixed-effects regression for longitudinal effects of independent variables on THB trajectory (g/L) (*n* = 100).

Variables	β	95% CI	*p* Value
Intercept	13.56	13.10, 14.02	<0.001
Group (adult)	−0.67	−1.28, −0.06	0.033
Time 1-Baseline	−0.86	−1.43, −0.28	0.003
Time 2-baseline	−0.49	−1.06, 0.08	0.092
Race (white)	−0.55	−1.13, 0.04	0.067
SRH (poor)	−0.44	−0.94, 0.07	0.090
Planned pregnancy (Yes, entirely)	0.39	0.02, 0.75	0.039
Group (adult) × Time(T1)	0.47	−0.33, 1.26	0.252
Group (adult) × Time(T2)	0.46	−0.33, 1.26	0.255
Group (adult) × Race (white)	1.01	0.23, 1.80	0.012

THB: Total hemoglobin. SRH: Self-reported health.

**Table 5:** Multivariate mixed-effects regression for longitudinal effects of independent variables on THB trajectory for stratified sample by age (*n* = 100).

	Adolescents		Adults	
Variables	β (95% CI)	*p*	β (95% CI)	*p*
Intercept	13.62 (13.09, 14.15)	<0.001	12.83 (12.36, 13.31)	<0.001
Time 1-baseline	−0.86 (−1.42, −0.30)	0.003	−0.40 (−0.95, 0.15)	0.158
Time 2-baseline	−0.48 (−1.04, 0.08)	0.092	−0.03 (−0.59, 0.52)	0.903
Planned pregnancy (yes)	0.18 (−0.43, 0.79)	0.560	0.57 (0.11, 1.02)	0.015
Race (white)	−0.56 (−1.23, 0.11)	0.100	0.46 (−0.01, 0.93)	0.057
SRH (poor)	−0.28 (−1.06, 0.51)	0.486	−0.58 (−1.22, 0.06)	0.075

THB: Total hemoglobin. SRH: Self-reported health. Note: Baseline refers to the evaluation up to the first 16 weeks of pregnancy, Time 1 to the third trimester of pregnancy and Time 2 to the 4–6 weeks postpartum.

## Data Availability

The dataset analyzed during the current study is available from the corresponding author on reasonable request.

## References

[1] Fisher A., Nemeth E. (2017). Iron homeostasis during pregnancy. Am. J. Clin. Nutr..

[2] Scholl T.O. (2005). Iron status during pregnancy: Setting the stage for mother and infant. Am. J. Clin. Nutr..

[3] Tan E.K., Tan E.L. (2013). Alterations in physiology and anatomy during pregnancy. Best Pract. Res. Clin. Obstet. Gynaecol..

[4] Jorgensen J.M., Crespo-Bellido M., Dewey K.G. (2019). Variation in hemoglobin across the life cycle and between males and females. Ann. N. Y. Acad. Sci..

[5] Bakrim S., Motiaa Y., Ouarour A., Masrar A. (2018). Hematological parameters of the blood count in a healthy population of pregnant women in the Northwest of Morocco (Tetouan-M’diq-Fnideq provinces). Pan Afr. Med. J..

[6] Dombrowski J.G., Barateiro A., Peixoto E.P.M., Barros A.B.C.S., Souza R.M., Clark T.G., Campino S., Wrenger C., Wunderlich G., Palmisano G. (2021). Adverse pregnancy outcomes are associated with Plasmodium vivax malaria in a prospective cohort of women from the Brazilian Amazon. PLOS Negl. Trop. Dis..

[7] Zillmer K., Pokharel A., Spielman K., Kershaw M., Ayele K., Kidane Y., Belachew T., Houser R.F., Kennedy E., Griffiths J.K. (2017). Predictors of anemia in pregnant women residing in rural areas of the Oromiya region of Ethiopia. BMC Nutr..

[8] Chaparro C.M., Suchdev P.S. (2019). Anemia epidemiology, pathophysiology, and etiology in low- and middle-income countries. Ann. N. Y. Acad. Sci..

[9] Fujimori E., Sato A.P.S., Szarfarc S.C., Veiga G.V., Oliveira V.A., Colli C., Moreira-Araújo R., de Arruda I.K.G., Uchimura T.T., Brunken G.S. (2011). Anemia in Brazilian pregnant women before and after flour fortification with iron. Rev. Saúde Pública.

[10] Holness N. (2015). A global perspective on adolescent pregnancy. Int. J. Nurs. Pract..

[11] Gibbs C.M., Wendt A., Peters S., Hogue C.J. (2012). The impact of early age at first childbirth on maternal and infant health. Paediatr. Perinat. Epidemiol..

[12] Ministry of Health of Brazil (2019). Information System on Live Births (SINASC), Single Health System Department of Informatics (DATASUS). http://tabnet.datasus.gov.br/cgi/tabcgi.exe?sinasc/cnv/nvuf.def.

[13] The World Bank Group (2021). Adolescent Fertility Rate (Births per 1000 Women Ages 15–19). https://data.worldbank.org/indicator/sp.ado.tfrt.

[14] Berhe B., Mardu F., Legese H., Gebrewahd A., Gebremariam G., Tesfay K., Kahsu G., Negash H., Adhanom G. (2019). Prevalence of anemia and associated factors among pregnant women in Adigrat General Hospital, Tigrai, northern Ethiopia, 2018. BMC Res. Notes.

[15] Ampiah M.K.M., Kovey J.J., Apprey C., Annan R.A. (2019). Comparative analysis of trends and determinants of anaemia between adult and teenage pregnant women in two rural districts of Ghana. BMC Public Health.

[16] Câmara S.M.A., Sentell T., Bassani D.G., Domingues M.R., Pirkle C.M. (2019). Strenghtening health research capacity to address adolescent fertility in Northeast Brazil. J. Glob. Health.

[17] Nunnally J., Bernstein I. (1994). Psychometric Theory.

[18] O’Broin S.D., Gunter E.W. (1999). Screening of folate status with use of dried blood spots on filter paper. Am. J. Clin. Nutr..

[19] McDade T.W. (2014). Development and validation of assay protocols for use with dried blood spot samples. Am. J. Hum. Biol..

[20] Qualtrics Software. https://www.qualtrics.com.

[21] Petruccelli J.L., Saboia A.L. (2014). Ethnic-Racial Characteristics of the Population: Classifications and Identities.

[22] Ministry of Health of Brazil (2020). Bolsa Família Program. https://bfa.saude.gov.br/public/file/faq_bfa.pdf.

[23] Andrade R.B., Pirkle C.M., Sentell T., Bassani D., Domingues M.R., Câmara S.M.A. (2020). Adequacy of Prenatal Care in Northeast Brazil: Pilot Data Comparing Attainment of Standard Care Criteria for First-Time Adolescent and Adult Pregnant Women. Int. J. Women’s Health.

[24] Food and Agriculture Organization of the United Nations (2012). Latin American and Caribbean Scale of Food Safety (ELCSA): User Manual and Applications. ELCSA Scientific Committee. http://www.fao.org/3/a-i3065s.pdf.

[25] Churchill D., Nair M., Stanworth S.J., Knight M. (2019). The change in haemoglobin concentration between the first and third trimesters of pregnancy: A population study. BMC Pregnancy Childbirth.

[26] Cao C., O’Brien K.O. (2013). Pregnancy and iron homeostasis: An update. Nutr. Rev..

[27] Cecchi F., Pancani S., Vannetti F., Boni R., Castagnoli C., Paperini A., Pasquini G., Sofi F., Molino-Lova R., Macchi C. (2017). Hemoglobin concentration is associated with self-reported disability and reduced physical performance in a community dwelling population of nonagenarians: The Mugello Study. Intern. Emerg. Med..

[28] Semasaka J.P.S., Krantz G., Nzayirambaho M., Munyanshongore C., Edvardsson K., Mogren I. (2016). Self-reported pregnancy-related health problems and self-rated health status in Rwandan women postpartum: A population-based cross-sectional study. BMC Pregnancy Childbirth.

[29] Kekäläinen P., Juuti M., Walle T., Laatikainen T. (2016). Pregnancy planning in type 1 diabetic women improves glycemic control and pregnancy outcomes. J. Matern.-Fetal Neonatal Med..

[30] Lee S.Y., Pearce E.N. (2021). Preconception care to Optimize Pregnancy Outcomes. JAMA.

[31] Chiossi G., Palomba S., Costantine M.M., Falbo A.I., Harirah H.M., Saade G.R., La Sala G.B. (2019). Reference intervals for hemoglobin and hematocrit in a low-risk pregnancy cohort: Implications of racial differences. J. Matern.-Fetal Neonatal Med..

[32] Mohamed M.A., Ahmad T., Macri C., Aly H. (2012). Racial disparities in maternal hemoglobin concentrations and pregnancy outcomes. J. Perinat. Med..

